# How Different Personalities Affect the Reaction to Adoption of Dogs Adopted from a Shelter

**DOI:** 10.3390/ani11061816

**Published:** 2021-06-18

**Authors:** Sara Corsetti, Luisa Pimpolari, Eugenia Natoli

**Affiliations:** 1 School of Agriculture and Environment, The University of Western Australia, Crawley, WA 6009, Australia; sar.corsetti@gmail.com; 2 Trinseo Italia Srl, Viale Certosa, 2, 20155 Milano, Italy; pimpa10.10@gmail.com; 3 Canile Sovrazonale, ASL Roma 3 (Local Health Unit Rome 3), via della Magliana 856H, 00148 Rome, Italy

**Keywords:** dog, shelter, behavioral differences, adoption

## Abstract

**Simple Summary:**

The adoption of dogs from shelters is very common in western societies, especially in countries where a ‘no-kill’ policy is enforced by law, like Italy. Many studies have focused on unsuccessful adoptions but few studies have aimed to evaluate the behavior of adopted dogs when adoption is successful and dogs remain with their new owners. This study aims to detect if there are behavioral modifications in dogs after successful adoption, based on their personality traits. We assessed the personality of 34 healthy dogs when housed in a shelter and, after a few years, the behavior of the same dogs was analyzed in their new house. The personality of the dogs affected their reaction to adoption: bolder dogs became even more active and playful. Changes were correlated to time since adoption. Considering that 100% of the dog adoptions in this study have been successful, the added value of this research is to help dog counselors who work in shelters to provide more focused suggestions for new owners who are adopting a dog.

**Abstract:**

Dog shelters provide a valuable service by housing homeless dogs and seeking subsequent adoption for these dogs. Few studies have aimed to monitor the behavior of adopted dogs when adoption is successful. The aim of this study was to detect what behavioral modifications, based on their personality, occurred in dogs after their adoption. The personality of 34 healthy dogs was evaluated in the pre-adoption phase by means of a Principal Component Analysis (PCA) of their behavioral patterns. In the post-adoption phase, we analyzed the behavior of the same dogs, completing a questionnaire with their owners. Pre- and post-adoption data were standardized and a PCA was run on the differences between these variables. A k-means cluster analysis was run on the six components, obtaining three groups of dogs: for groups one and two, changes in behavior after adoption seemed to be influenced by dog personality: bolder dogs (1st group) became more active, excitable and playful, showed increased aggressive behavior towards humans, and decreased anxious and submissive behavior towards dogs and humans; shyer dogs (2nd group) went in the opposite direction, displaying increased aggressive behavior. For the 3rd group, personality was not predictive of behavior changes. All the dog adoptions in this study were successful.

## 1. Introduction

Dog shelters are common in westernized societies, especially in Italy where a “no-kill” policy has been enforced by law since 1991 (some crucial points of Italian Law 281/1991 are: (1) unowned free-roaming dogs cannot be euthanized unless they are incurable or proven to be dangerous; (2) unowned free-roaming dogs must be captured and taken to a dog shelter; (3) all municipalities must have a dog shelter; (4) all dogs in the shelters must be registered and neutered; (5) all owned dogs have to be registered; (6) shelter dogs can be adopted; and (7) shelter dogs cannot be used as experimental animals in scientific research.). In our country, shelters provide a valuable service by hosting unowned free-roaming dogs (unowned free-roaming dogs includes all dogs without an owner who wander, exploit human waste-food resources, or live on hunted prey) [1], dogs brought in by their owners for various reasons (e.g., aggressive behavior towards people and other animals, disobedience, anxiety disorders and/or a combination of these reasons; Natoli unpublished, according to what has been found in other countries [2,3]), and dogs seized by the Public Veterinary Service because of maltreatment by former owners. The relevant Italian law (Law 189/2004) provides for the seizure of the dog by the Local Health Unit responsible for that territory, for cases in which dogs are confined to inadequate spaces, denied appropriate access to food or water, denied physical exercise or veterinary care, exposed to torture leading to injury, or exposed to working conditions or experiences that result in fatigue or injury or do not meet the animals’ ethological needs. Such local units also seek subsequent adoption for impounded dogs.

In Italy, the adoption of dogs from shelters, in particular from public ones, is widespread, especially in large cities. In the Muratella municipal dog shelter in Rome, for example, which housed from 400 to 500 dogs in the last three years, the majority of dogs are adopted within one year of their arrival at the shelter [1]. There is a low percentage of relinquishments from new owners: from 2017 to 2019, 5.62% of adopted dogs came back to the Muratella shelter (dog shelter database, personal communication).

Most studies in this area of research have focused on the factors that bring about the relinquishment of dogs adopted from shelters (reviewed in [4]), but few studies have aimed to monitor the behavior of adopted dogs when adoption is successful and dogs remain with their new owners (see [5]). Even fewer successful adoptions are monitored over the long term. Thus, if it is interesting to verify how a dog copes with their entrance into a stressful environment, such as the shelter [6], it is equally interesting to verify how a dog copes with their exit from the shelter once adopted and following entry into an unknown house. Although the second event is always viewed in positive terms, it can still be a stressful event. In causal terms, a stressful event is defined as any occurrence that changes a living environment, making it unpredictable, and that can be accompanied by anxiety and fear [7]. For a dog, moving into an unknown house might be a stressful event because of the unfamiliarity of new people and the new home [8].

It has been shown that individuals, even of the same species, differ from each other in behavior and underlying physiology when having to cope with challenges in their environment, both social and non-social. These individual differences are called personality traits or dimensions. Personality, therefore, is a cluster of psychological and behavioral traits that are interrelated; they differ as a whole from one individual to another but within the same individual they are quite stable over time [9,10,11,12,13,14,15,16]. However, it has also been shown that, despite its rigidity [17,18], personality is endowed with a substantial phenotypic plasticity that can be influenced by environmental factors [19,20,21,22], thus determining how an individual modifies their behavior in response to changes in environmental conditions [23]. Certainly, personality determines how an individual copes with a new environment [10].

Dog personality has been extensively studied and several methods have been suggested for its assessment (reviewed in [24]). For example, test batteries have been utilized to gather data from different test situations and evaluate dogs using well-defined and directly observable behavior units (in terms of duration, frequency, and latencies). Another approach is to evaluate the results of test batteries utilizing behavioral ratings that, although carried out by an expert in dog behavior, are of course less objective. Finally, in their review, Miklosi et al. [24] described a third method for assessing personality, i.e., collecting behavioral data via questionnaires filled in by owners. This last method has the advantage of ease of administration, and its external validity (at least with respect to dominance rankings) has recently been verified [25].

The aim of this study was to document dogs’ behavioral reactions to a complete change in their environment as a function of their personalities, since it has been found that dogs are routine-based animals [26]. To achieve this objective, the behavior of the dogs under study was observed in the shelter prior to adoption and their personality assessed. Subsequently, after a few years, the behavior of the same dogs was analyzed by means of a questionnaire administered to the owner after adoption. We hypothesized that all dogs would show changes in behavior after adoption but that the nature of those changes would be influenced by their personality (as assessed in the shelter prior to adoption).

## 2. Materials and Methods

### 2.1. Animals and Housing

The subjects of this study were 34 healthy dogs: 17 females and 17 males, 27 mixed-breed and 7 phenotypically purebred-derived dogs (four Maremma Shepherds, two Groenendael Belgian Sheperds and two German Shepherds), with ages ranging from one to six years when they were observed in the pre-adoption phase of the study. All of these dogs were middle-size and gonadectomized at least one month before the behavioral observations began (Italian law # 128 (1991) requires that all dogs in shelters are neutered).

None of them were particularly aggressive; in other words, the dogs chosen for the study did not display warning signs that fall into the category of aggressive behavior, such as growling and snarling, nor did they attack by lunging and biting. They did not show any behavior patterns that would prevent them from being allowed to interact freely with humans. Such dogs were selected as subjects because several of the behavioral observations to be made required that the dogs interact with observers in the pre-adoption phase (see [26]), interaction which is impossible with aggressive individuals. In addition, dogs showing signs of severe aggressive behavior would never be offered for adoption following the strict rules for adoption in shelters.

In the pre-adoption phase the dogs were housed in a Public Shelter and Veterinary Hospital called Porta Portese, situated in Rome; it was a long-standing dog shelter, where dogs were kept in pairs or alone in small cages (see [26]).

In the post-adoption phase the same 34 dogs were homed: 3 of them lived with a single owner, whereas 31 dogs lived in a family with between two and five human beings. When this study was carried out, the period since dogs’ adoptions ranged from two to four years.

At the beginning of the post-adoption phase, when the second part of the study began (beginning of 2005), 100% of the adopting owners reported being satisfied with their adopted dog, despite the occurrence of some unwanted behaviors reported in a low percentage (<10%) of respondents (e.g., inappropriate evacuation elimination behavior not linked to territorial marking behavior or the habit of chewing objects).

### 2.2. Behavioral Observations

In the pre-adoption phase, data were collected by three previously trained observers, whose inter-observer reliability ranged from 0.82 to 0.89 (the value of agreement between observers deemed acceptable to avoid biased results is 0.75). The behavioral observations were performed by a single observer each time who sat in front of the cage using the focal animal sampling method (one dog each time), with ‘All occurrences’ and ‘1/0′ methods (60 s interval) [27]; the ethogram, based on the study of De Palma et al. [26], consisted of 111 behavioral patterns (Appendix A), gathered into 12 categories (Table 1) on the basis of the evidence conventionally utilized in ethology (see, for example, [28]). Evidence is of two types: contextual and consequential. The former is based on the fact that some behaviors are observed in some contexts but not in others and therefore have a function related to those contexts, while the other criterion is based on grouping together behaviors that have the same function [28]. These criteria have been used in previous studies [6,26,29,30].

Each dog was observed for 30 min on three different days, for a total of 1.5 h.

In the post-adoption phase, to evaluate the behavior of the same 34 dogs after they had been adopted and rehomed, we contacted owners by telephone (March to October 2005). If the owner consented to participate, an observer went to their house to interview them using a pre-made questionnaire. The questionnaire (described in more detail in Appendix B) was developed to try to collect data regarding the same behaviors that had been used to determine dog personality when in the shelter. Each interview lasted about two hours. Eight participants did not consent to be visited in their homes; for these participants, interviews were conducted by telephone.

Despite the fact that it would have been better to collect data in the same way as the data were collected in the shelter, such a long visit in a private house was unthinkable. Actually, in order to obtain behavioral information on pet dogs, we consider the questionnaire administered to owners as a compromise, since it is impossible to avoid a person’s subjectivity when judging their own dog. For this reason, we made up our own very simple questionnaire with a few questions useful for obtaining scores for the behavioral categories utilized in the pre-adoption phase. Thus, the questionnaire was created by trying to reflect the ethological observations carried out in the shelter during the first phase of the study; to reach this goal, we had to pay the price of utilizing a non-validated questionnaire.

The questionnaire consisted of 12 questions, each of which referred to one behavioral category utilized during the pre-adoption phase observation in the shelter. For each question, owners were asked to assign a score from 1 to 5 (1 = never, 2 = almost never or rarely, 3 = sufficiently, on average, 4 = usually, almost always, 5 = always, a lot) (Appendix B).

Another unavoidable limitation of the study was that the new homes differed one from the other. Of course, this variability must have influenced dog behavior changes but we still think it was worthwhile to be measured.

### 2.3. Statistical Analysis

In the pre-adoption phase, a Principal Component Analysis (PCA) was run on the 12 behavioral categories (Table 1) utilized as variables. The basic goal of the PCA was to reduce the dimensionality of a data set while retaining as much information as possible. The analysis replaced the original correlated variables with new uncorrelated component variables called principal components. The analysis ordered them using as criterion the decreasing amounts of contribution to the sum of the variances of the original variables (the eigenvalues). So, a small number of principal components, linear combinations of the initial variables, was kept and explained most of the variation in the data. The first principal component was the combination of variables that accounted for the maximum amount of variation. The second principal component accounted for the next largest remaining amount of variation and was orthogonal to the first principal component.

The number of components of the PCA run on frequency of dog behavioral patterns recorded in the shelter was two, and were chosen with the aid of the ‘Parallel Analysis Engine’ [31] which calculates eigenvalues from randomly generated correlation matrices. These can then be compared with the eigenvalues extracted from the researcher’s dataset (in this case our dataset). The number of components retained was chosen on the basis of the number of eigenvalues (generated from the researcher’s dataset) that are larger than the corresponding random eigenvalues.

In addition, PCA further transformed the information on the dogs into individual scores for each component, adjusting the variances in a similar way to the behavioral variables. Thus, the two aspects of the PCA are both important. In summary, the PCA reduced the 12 original behavioral variables to two components that described them all, indicating whether these go in the same direction or not in an orthogonal plane and, at the same time, computed an individual score for each of the two components per dog. Therefore, the PCA allowed the characterization of the animals by common or opposite behaviors and was a way of building a typology of individual.

The PCA components were named based on the variables with which they showed correlations.

Concerning the comparison of the two periods, in order to highlight any change in dog behavior each variable of the pre- and post-adoption data was standardized (value minus mean/standard deviation) and the difference in this data was calculated for each dog (standardized post-adoption score for each category minus standardized pre-adoption score for each category). A Principal Component Analysis (PCA) was run on these variables, i.e., on the differences in the scores (standardized), yielding 6 components. The changes were calculated as post- minus pre-adoption and they can be interpreted directly as variations: a positive score indicates an increase in that component and a negative score indicates a decrease in that component.

A k-means cluster analysis was run on the six components (compared using the mean of a one factor ANOVA), obtaining three groups of dogs.

Since the dogs were assigned to the three groups highlighted by the cluster analysis because of the different changes in their behavior during the post adoption period, a one factor ANOVA was performed to explore the possibility that the changes were influenced by the individual differences detected during the pre-adoption phase (cluster assigned group = independent variable; individual factor one (F1) score and factor two (F2) score = dependent variable).

To determine whether or not group assignment varied by sex, a chi-square analysis was performed.

To check if there was a correlation between the time that had passed since a dog was adopted from the shelter (no. of years), and individual score differences in post-adoption minus pre-adoption behaviors, a Spearman’s correlation test was employed.

All statistical analyses were carried out using SPSS Statistics (IBM Corp^®^, Armonk, NY, USA; release 25).

## 3. Results

The first two components of the PCA run on the pre-adoption data explained 40.14% of the total variance. For all components, any correlation of 0.50 or above was deemed relevant for the variable loading (Table 2). The first component (F1) was defined as “insecurity” since, along the axis, it was highly correlated with measures of “anxiety” and with measures of “subordination and aggressive behavior”. Thus, dogs with high F1 positive values were shy individuals because they were aggressive and with subordinate attitudes; dogs with high F1 negative values were anxious. The second component (F2) was defined as “self-confidence” since it was highly correlated with measures of “dominance towards dogs, attention and affiliative behavior towards humans” and with measures of “playfulness”. Thus, dogs with high F2 positive values were bold individuals because they were attentive, sociable towards humans, and with dominant attitudes towards dogs; dogs with high F2 negative values were also playful.

The first six components of the PCA on the difference between pre- and post-adoption data (Table 3) explained 77% of the total variance. For all components, any correlation of 0.62 or above was deemed relevant for the variable loading (Table 3). The PCA shows that, after adoption, there was an increase in activity, excitability, and playfulness (1st component), in subordination towards humans and dogs (2nd component), in aggressiveness towards dogs (3rd component), in affiliative behavior towards humans and in attention (4th component), in aggressiveness towards humans (5th component) and in dominance towards dogs (6th component); there was also a decrease in affiliative behavior towards dogs (3rd component) and anxiety (5th component). The cluster analysis highlighted three groups of 6, 8 and 20 dogs (Table 4) based on their behavioral changes after adoption; the groups differed in all components except than for the 3rd and 4th (one factor ANOVA. 1st component: *F*_2,31_ = 12.3, *p* = 0.0001; 2nd component: *F*_2,31_ = 5.11, *p* = 0.012; 3rd component: *F*_2,31_ = 0.61, *p* = 0.548; 4th component: *F*_2,31_ = 2.35, *p* = 0.112; 5th component: F_2,31_ = 3.54, *p* = 0.041; 6th component: *F*_2,31_ = 5.06, *p* = 0.013).

It seems that the behavioral characteristics of the dogs recorded when the dogs were in the shelter determined their cluster group. The results of the one factor ANOVA (where the cluster assigned group is the independent variable and the individual F2 score is the dependent variable) suggested that there was a slight prevalence of self-confident dogs in group 1, all shy dogs were in group 2, whereas dogs in group 3 were spread all over the gradient (*F*_2,31_ = 6.37, *p* = 0.005). In contrast, the one factor ANOVA where the cluster assigned group was the independent variable and the individual F1 score was the dependent variable was not significant (1st factor: *F*_2,31_ = 1.30, *p* = 0.288).

The results suggest that after their adoption, 26 dogs out of 34 were more active, excitable and playful. This change was mainly due to the dogs belonging to the 1st and 3rd group. In contrast, the 8 dogs belonging to the 2nd group, wherein the dogs were classified as shyer, decreased these displays. Submissive behavior, both towards humans and other dogs, remained almost the same in the shyer dogs while it decreased in the six dogs of the first group and increased in the 20 dogs belonging to the third group. Aggressive behavior towards humans increased in the 14 dogs belonging to the 1st and 2nd group, but decreased in the 20 dogs belonging to the 3rd group; the opposite occurred for anxious behavior. Dominant behavior towards other dogs increased after the adoption, but mainly in the 3rd group dogs.

There was no effect of sex on the group in which the dogs clustered (*χ*^2^ = 0.70, df = 2, NS).

The time elapsed since the adoption and the difference in the individual “before-after” adoption scores were not highly correlated for the six components of the 1st (Spearman rank correlation test, two tailed: rho = −0.349, *n* = 34, *p* = 0.043) and 2nd (rho = 0.444, *n* = 34, *p* = 0.009) factors; however, the correlation, although significant, was not particularly high. In other words, the results show that the longer was the time which had elapsed since the adoption, the smaller was the increase in dog activity, excitability and playfulness (i.e., the frequency of these behaviors increased soon after the adoption) and the other way round: the greater was the increase in dog subordination towards human beings and other dogs (i.e., the frequency of the latter increased with the time elapsed).

## 4. Discussion

The results of this study suggest that dogs adopted from a dog shelter, after a few years from the time of their adoption, show behavioral changes in their new house that may have been influenced by their personality, as assessed when the dogs were still in the shelter.

Determining a dog’s personality in a dog shelter environment is a challenge not easy to deal with, because the variables that can influence the behavior of dogs are significant; in addition to the more intuitive ones, such as the availability of space, housing alone or with other dogs, the frequency of contact with human beings, the frequency of going out from the cage and so on [32]; for example, McGuire et al. [33] found that, in a shelter, even the sex of the walker influences dog’s elimination behavior. Furthermore, the structure is not designed to perform behavioral test batteries properly. This is the reason why we decided, in this study and in other studies before [26], to evaluate the dog’s personality by processing the data collected by means of standard ethological techniques [27]: through direct observation of their behavior. This choice was due to the belief that applying standardized behavior tests to dogs with dissimilar backgrounds does not produce meaningful results. The reaction of a dog born free-ranging may not be comparable to that of a dog born as a pet and then later relinquished to the shelter as an adult. This point of view has also been endorsed by others (see, for example, [34]). The direct observation of animals is less susceptible to subjectivity or errors due to the lack of homogeneity of the sample (different dog backgrounds) or to an environment not designed for research, and it has been utilized for some species of non-human primates (chimpanzees (*Pan troglodytes*) [35,36]; olive baboons (*Papio anubis*) [37]; bonobos (*Pan paniscus*) [38]).

Unfortunately, as specified in the methods, in the second part of the study we were unable to conduct the behavioral observations of the dogs with the same methods in the owners’ homes; this would have resulted in too long a stay in private homes and an imposition of rules (for example not to interact with their dog) which are very difficult to respect. Thus, we utilized a very simple questionnaire provided to the owners to evaluate the dog’s personality. Although in this way the answers may have been influenced by the owner’s subjectivity, we have tried to limit this danger by presenting the questionnaire directly to the owners in person, and corroborating it with a wealth of information aimed at eliminating misunderstandings.

We want to highlight that the aim of this study was not to investigate the predictive value of personality assessment tests utilized in the shelter on the likelihood that the dogs showed problematic behavior after adoption, as in [5,39]. We aimed to detect which behavioral changes, within the behavioral repertoire of the species, occurred in the new house.

The results of this paper in terms of detecting changes in the behavior of dogs in the two environments, pre- and post-adoption, are not surprising in themselves: the two environments are fundamentally different (dog shelter vs. home of the owner), therefore the expression of some behaviors will be obviously different. What can be interesting is that the behavioral changes of the dogs might have been influenced by their personality.

Although individual personality traits are considered fairly stable across time and context [9,10,11,15,16,17,18,19], the results of this paper might support the view that personality has a substantial phenotypic plasticity: the latter emerges when an individual modifies their behavior because they are forced into a completely new environment [23].

Such environmental factors include changes in the context in which a trait is expressed, for example, and the social factors that will change the adaptive value of a certain behavioral response (e.g., [40]).

However, the behavioral changes in the dogs showed an inherent logic: the shyer dogs became less active, excitable, and playful although more alert, as is logical to expect from an individual who tends to adapt to their environment rather than manipulate it; they did not change their submissive behavior toward humans nor their submissive or dominant behavior toward other dogs, as would be expected from a shy individual; their aggressive behavior towards other dogs decreased, their affiliative behavior at the intra- and inter-specific level increased, and they became less anxious; the last changes were probably due to the increase in their self-confidence. Unfortunately, they became more aggressive towards humans and this change prevents us from saying that their management had become easier.

In contrast, bolder dogs increased their activity, excitability, and playfulness, and generally decreased their attention to their environment, as might be expected based on their bold personality traits; moreover, they showed less inter- and intra-specific submissive behavior, and they were less anxious but more aggressive toward humans, as one might expect when a dog becomes conscious of having obtained important resources like food and refuge.

On the one hand, the time elapsed from adoption played a role in decreasing the tendency for dogs to be active, excitable and playful, although this can be due also to the increase in the age of the dogs. On the other hand, the positive correlation between time occurred since adoption and the individual score for the 2nd factor of the PCA on the difference between post- and pre-adoption suggested that the longer the dogs were adopted, the higher was the increase in their submissive behavior, which could be due to habitually respecting the rules given by an owner.

## 5. Conclusions

The management of a dog in a shelter is a challenge, especially in Italy where the no-kill policy is enforced by law. The desirable outcome for each single dog is adoption. Despite the large number of studies, too little is known about the characteristics that condition adoption success or failure. Considering that 100% of the dog adoptions in this study have been successful, the added value of this research is to provide additional information to help dog counsellors who work in shelters provide more focused suggestions to new owners who are adopting a dog.

## Figures and Tables

**Table 1:** The behavioral categories utilised for the PCA as variables.

Behavioral Category	Observed Behavioral Patterns
Activity	Standing, walking, or trotting in/out from the internal to the external area of the cage and vice-versa.
Aggressive behavior towards humans	Growling, sideways glances, raising fur, curling lips, showing teeth, dashing at bars.
Aggressive behavior towards dogs	Growling, sideways glance, raising fur, curling lip, showing teeth, dashing at bars.
Anxiety	Body shaking, scratching, muzzle licking, self-grooming, yawning, repetitive pacing in circles, licking or biting compulsively, catching flies, coprophagy, self-mutilation.
Attention	Raising ears, looking outside, looking out carefully, looking at observer, looking at unknown people, looking at volunteer, looking at dog, raising foreleg, raising forelegs on wall, sniffing environment, sniffing air, sniffing observer, sniffing unknown people, sniffing volunteer, sniffing dog.
Dominant behavior towards dogs	Staring, stiff body and tail still, raised tail, wagging with the tail held high, pricked-up ears, urinating with a raised leg, paw or a muzzle on a conspecific’s back.
Submissive behavior towards humans	Avoiding eye contact, lowering head, ears down, cringing, tail between the legs, lying down on the back.
Submissive behavior towards dogs	Avoiding eye contact, lowering head, ears down, cringing, tail between the legs, lying down on the back.
Excitability	Drooling, try to mount, pointer, try to chase a cat, galloping, jumping.
Affiliative behavior towards humans	Wagging tail, giving the foreleg, leaning on bars.
Affiliative behavior towards dogs	Wagging tail, licking the muzzle of a dog, passive contact, allo-grooming.
Playing	Inviting to play, answering invitation to play.

**Table 2:** Results from the PCA run on pre-adoption data.

	Factor 1	Factor 2
	Insecurity	Self Confidence
Submissive towards dogs	0.840 *	−0.234
Submissive towards humans	0.823 *	−0.037
Aggressive towards humans	0.792 *	0.061
Anxiety	−0.570 *	−0.286
Aggressive towards dogs	0.532 *	−0.066
Excitability	−0.347	0.040
Activity	−0.340	0.207
Dominance towards dogs	−0.261	0.706 *
Attention	0.040	0.685 *
Affiliative towards humans	−0.065	0.585 *
Playfulness	−0.164	−0.515 *
Affiliative towards dogs	−0.084	0.298

*—Loadings > 0.50.

**Table 3:** Results from the PCA run on the difference between post- and pre-adoption data.

Behavioral Categories	Components					
	1	2	3	4	5	6
Submissive towards humans	−0.125	0.815 *	0.024	−0.063	0.106	−0.046
Submissive towards dogs	−0.221	0.626 *	−0.010	−0.224	0.068	−0.475
Aggressive towards humans	−0.183	0.176	0.317	−0.303	0.632 *	−0.128
Aggressive towards dogs	−0.036	0.399	0.692 *	−0.116	0.377	−0.057
Dominance towards dogs	0.004	−0.160	−0.109	−0.001	−0.057	0.896 *
Activity	0.761 *	−0.024	−0.085	0.264	−0.075	0.007
Excitability	0.626 *	−0.425	0.318	0.168	0.272	0.148
Affiliative towards humans	0.183	0.390	0.048	0.632 *	0.049	0.488
Affiliative towards dogs	0.037	0.158	−0.867 *	−0.055	0.212	0.077
Anxiety	−0.082	−0.023	0.207	−0.213	−0.848 *	−0.004
Playfulness	0.853 *	−0.117	−0.078	−0.224	−0.034	0.039
Attention	−0.008	−0.357	−0.021	0.833 *	0.038	−0.041

*—Loadings > 0.62.

**Table 4:** Results from the Cluster analysis.

	Cluster		
	1	2	3
Component 1: Activity, excitability, playfulness	0.55800	−1.16899	0.30020
Component 2: Submissive behavior	−1.00230	−0.06218	0.32556
Component 3: Aggressive towards dogs, affiliative towards dogs (opposite sign)	0.40421	−0.16293	−0.05609
Component 4: Affiliative towards humans, attention	−0.49745	0.58338	−0.08412
Component 5: Aggressive towards humans, anxiety (opposite sign)	0.32692	0.62147	−0.34666
Component 6: Dominance towards dogs	−1.02936	0.03229	0.29589

## Data Availability

Data available on request.

## Appendix A

**Table A1:** The Ethogram Utilised in This Study for Behavioural Observations during the Pre-Adoption Phase (De Palma et al. 2005).

Behaviour	Description of the Behaviour
***Activity***	
Standing	staying in an upright position, on four legs.
Walking	walking in the cage.
Trotting	trotting in the cage.
In/out	going in and out of the indoor/outdoor zone of the cage.
***Aggressive behaviour***	
Growling	threatening vocalisation coming from the throat.
Sideways glance	looking transversely with the head upright or bent. The glance is threatening.
Raising fur	raising the fur of the head, body and tail so that the dog appears to have a larger size and is thus more threatening.
Curling lip	light raising of the upper lip, usually only on one side, with a threatening partial display of the teeth.
Showing teeth	curling of the upper and lower lips while opening the mouth with a threatening display of the teeth.
Dashing at bars	dashing at bars in the direction of the observer, of another person or of another dog.
***Anxiety***	
Body shaking	shaking the body quickly sideward.
Scratching	raising one hind leg and vigorously scratching part of the body with the leg.
Muzzle licking	passing the tongue over the muzzle.
Self-grooming	cleaning itself with the tongue and the teeth.
Yawning	opening the mouth and inhaling and exhaling air.
Repetitive pacing in circles	repetitive walking in a circle within the cage.
Licking or biting compulsively	repeatedly licking or biting the bars, the wall and/or objects in the cage.
Catching flies	trying to catch an imaginary fly with the mouth, clutching at empty air with the teeth.
Coprophagy	eating feces.
Self-mutilation	licking itself continuously in same part of the body, so intensely to cause abrasions or even wounds.
***Attention***	
Raising ears	raising ears because dog’s attention has been caught by something.
Looking outside	looking outside the cage, across the bars.
Looking out carefully	looking outside the cage very carefully; the position resembles that described for "prompt" but the dog is not ready to spring up.
Looking at observer	looking outside the cage towards the observer.
Looking at unknown people	looking at people the dog does not know.
Looking at volunteer	looking at a shelter volunteer worker.
Looking at dog	looking outside the cage towards another dog.
Raising foreleg	raising one foreleg towards something or someone that caught dog’s attention.
Raising forelegs on wall	raising both forelegs onto the wall or onto the bars, looking carefully outside
Sniffing environment	putting the muzzle on the ground, on the wall, or on the objects in the cage, the dog sniffs the environment.
Sniffing air	raising the head, moving the nostrils and breathing the air to perceive odors.
Sniffing observer	pointing the muzzle towards the observer, the dog moves the nostrils clearly trying to perceive the odors of the observer.
Sniffing unknown people	pointing the muzzle towards people the dog does not know, the dog moves the nostrils clearly trying to perceive their odors.
Sniffing volunteer	pointing the muzzle towards volunteers working in the shelter, the dog moves the nostrils clearly trying to perceive their odors.
Sniffing dog	pointing the muzzle towards another dog, the subject moves the nostrils clearly trying to perceive the object’s odors.
***Dominant behaviour***	
Staring	gazing at the observer, another person or another dog right in the eyes.
Stiff body and tail still	standing still in an upright posture, with the ears raised and turned forward, the four legs straight and rigid and the tail immobile and rigid at a medium height.
Raised tail	the tail is held high while it is still.
Wagging with the tail held high	moving the tail sideward while held high.
Pricked-up ears	holding the ears forwards while assuming an upright body posture with head and tail held high, legs straight and stiff.
Urinating with a raised leg	urinating keeping a leg raised.
Paw/muzzle on a conspecific’s back	putting the muzzle or one forepaw or both over the back of another dog.
***Submissive behaviour***	
Avoiding eye contact	looking away from the observer, another human or another dog, who is looking at the subject.
Lowering head	lowering the head in front of the observer, another human or another dog.
Ears down	putting the ears down, pressed on the head, or holding them backwards.
Cringing	lying with the ventral region in contact with the ground.
Tail between the legs	holding the tail down or tightly between the hind legs and against the belly.
Lying down on back	laying down on the back exposing the ventral side of the chest and sometimes the abdomen.
***Excitability***	
Drooling	the dog flows saliva out of the mouth.
Try to mount	mounting another dog simulating a copulation.
Pointer	holding the ears forwards while assuming an upright body posture with head and tail held high, legs straight and stiff.
Try to chase a cat	pointing and showing the intention to chase a cat.
Galloping	galloping in the cage.
Jumping	jumping in the cage.
***Affiliative behaviour***	
Wagging tail	the tail is wagged sideward but not held high, in a relaxed manner.
Giving the foreleg	raising one of the forelegs and leaning it in the direction of the observer.
Leaning on bars	leaning the body in direct contact with the bars of the cage.
Licking the muzzle of a dog	licking the muzzle of other dogs.
Passive contact:	staying in contact with some part of the body, without sleeping.
Allo-grooming	cleaning the fur of another dog, licking and nibbling.
***Playing***	
Inviting to play	inviting another dog or human to play: the dog bends down with the forelegs outstretched on the ground and the rump upwards, or brings an object, runs around and jumps.
Answering invitation to play	playing with another dog after having been invited to do so.

## Appendix B

**Table A2:** The Questionnaire Utilized by the Owners to Describe Dog Behaviour.

1. Is the dog subordinate to humans?	1 2 3 4 5
2. Is the dog subordinate to dogs?	1 2 3 4 5
3. Is the dog aggressive towards humans?	1 2 3 4 5
4. Is the dog aggressive towards dogs?	1 2 3 4 5
5. Is the dog dominant towards dogs?	1 2 3 4 5
6. Is the dog active?	1 2 3 4 5
7. Is the dog excitable?	1 2 3 4 5
8. Is the dog sociable towards humans?	1 2 3 4 5
9. Is the dog sociable towards dogs?	1 2 3 4 5
10. Is the dog anxious?	1 2 3 4 5
11. Is the dog playful?	1 2 3 4 5
12. Is the dog attentive?	1 2 3 4 5

## References

[1] Natoli E., Cariola G., Dall’Oglio G., Valsecchi P. (2019). Considerations of ethical aspects of control strategies of unowned free-roaming dog populations and of the no-kill policy in Italy. J. Appl. Anim. Res..

[2] Lambert K., Coe J., Niel L., Dewey C., Sargeant J.M. (2015). Systematic review and meta-analysis of the proportion of dogs surrendered for dog-related and owner-related reasons. Prev. Vet. Med..

[3] Jensen J.B.H., Sandøe P., Nielsen S.S. (2020). Owner-Related Reasons Matter more than Behavioral Problems—A Study of Why Owners Relinquished Dogs and Cats to a Danish Animal Shelter from 1996 to 2017. Animals.

[4] Salman M.D., Hutchison J., Ruch-Gallie R., Kogan L., New J.C., Kass P.H., Scarlett J.M. (2000). Behavioral Reasons for Relinquishment of Dogs and Cats to 12 Shelters. J. Appl. Anim. Welf. Sci..

[5] Valsecchi P., Barnard S., Stefanini C., Normando S. (2011). Temperament test for re-homed dogs validated through direct behavioral observation in shelter and home environment. J. Vet. Behav..

[6] Corsetti S., Borruso S., Di Traglia M., Lai O., Alfieri L., Villavecchia A., Cariola G., Spaziani A., Natoli E. (2018). Bold personality makes domestic dogs entering a shelter less vulnerable to diseases. PLoS ONE.

[7] Sapolsky R.M., Romero M., Munck A.U. (2000). How do glucocorticoids influence stress responses integrating permissive, suppressive, stimulatory, and preparative actions. Endocr. Rev..

[8] Stephen J., Ledger R. (2007). Relinquishing dog owners’ ability to predict behavioural problems in shelter dogs post adoption. Appl. Anim. Behav. Sci..

[9] Carere C., Maestripieri D., The University of Chicago Press (2013). Animal Personalities: Behavior, Physiology, and Evolution.

[10] Sih A., Bell A., Johnson J.C., Ziemba R.E. (2004). Behavioral syndromes: An integrative overview. Q. Rev. Biol..

[11] Sih A., Bell A., Johnson C.J. (2004). Behavioral syndromes: An ecological and evolutionary overview. Ecol. Evol..

[12] Pervin L., John O.P. (1997). Personality: Theory and Research.

[13] Budaev S.V. (1997). “Personality” in the guppy (Poecilia reticulata): A correlational study of exploratory behavior and social tendency. J. Comp. Psychol..

[14] Gosling S.D. (2001). From mice to men: What can we learn about personality from animal research?. Psychol. Bull..

[15] Vazire S., Gosling S.D., Bekoff M. (2004). Personality and temperament: A comparative perspective. Encyclopedia of Animal Behavior.

[16] Bell A.M. (2007). Future directions in behavioural syndromes research. Proc. R. Soc. B Biol. Sci..

[17] Koolhaas J.M., Korte S.M., De Boer S.F., van der Vegt B.J., Van Reenen C.G., Hopster H., De Jong I.C., Ruis M.A., Blokhuis H.J. (1999). Coping styles in animals: Current status in behavior and stress physiology. Neurosci. Biobehav. Rev..

[18] Van Oers K., de Jong G., Van Noordwijk A.J., Kempenaers B., Drent P.J. (2005). Contribution of genetics to the study of animal personalities: A review of case studies. Behaviour.

[19] Carere C., Drent P.J., Koolhaas J.M., Groothuis T.G.G. (2005). Epigenetic effects on personality traits: Early food provisioning and sibling competition. Behaviour.

[20] Arnold K.E., Ramsay S.L., Donaldson C., Adam A. (2007). Parental prey selection affects risk-taking behaviour and spatial learning in avian offspring. Proc. R. Soc. Lond. B.

[21] Krause E.T., Honarmand M., Wetzel J., Naguib M. (2009). Early fasting is long-lasting: Differences in early nutritional conditions reappear under stressful conditions in adult zebra finches. PLoS ONE.

[22] Stamps J.A., Groothuis T.G.G. (2010). Developmental perspectives on personality: Implications for ecological and evolutionary studies of individual differences. Philos. Trans. R. Soc. Lond. B.

[23] Nussey D.H., Wilson A.J., Brommer J.E. (2007). The evolutionary ecology of individual phenotypic plasticity in wild populations. J. Evol. Biol..

[24] Miklosi A., Turcsan B., Kubinyi E., Kaminski J., Marshall-Pescini S. (2014). The personality of dogs. The Social Dog: Behavior and Cognition.

[25] Kubinyi E., Wallis L.J. (2019). Dominance in dogs as rated by owners corresponds to ethologically valid markers of dominance. Peer J..

[26] De Palma C., Viggiano E., Barillari E., Palme R., Dufour A.B., Fantini C., Natoli E. (2005). Evaluating the temperament in shelter dogs. Behaviour.

[27] Altmann S.A. (1974). Observational study of behaviour: Sampling methods. Behaviour.

[28] Troisi A. (1999). Ethological research in clinical psychiatry: The study of nonverbal behavior during interviews. Neurosci. Biobehav. Rev..

[29] Corsetti S., Ferrara M., Natoli E. (2019). Evaluating Stress in Dogs Involved in Animal-Assisted Interventions. Animals.

[30] Corsetti S., Borruso S., Malandrucco L., Spallucci V., Maragliano L., Perino R., D’Agostino P., Natoli E. (2021). Cannabis sativa L. may reduce aggressive behaviour towards humans in shelter dogs. Sci. Rep..

[31] Horn J.L. (1965). A Rationale and Test for the Number of Factors in Factor Analysis. Psychometrika.

[32] Cafazzo S., Maragliano L., Bonanni R., Scholl F., Guarducci M., Scarcella R., Di Paolo M., Pontier D., Lai O., Carlevaro F. (2014). Behavioural and physiological indicators of shelter dogs’ welfare: Reflections on the no-kill policy on free-ranging dogs in Italy revisited on the basis of 15 years of implementation. Physiol. Behav..

[33] McGuire B., Fry K., Orantes D., Underkofler L., Parry S. (2020). Sex of walker influences scent-marking behavior of shelter dogs. Animals.

[34] Patronek G.J., Bradley J. (2016). No Better Than Flipping a Coin: Reconsidering Canine Behavior Evaluations in Animal Shelters. J. Vet. Behav..

[35] Van Hooff J.A.R.A.M. (1973). The Arnhem Zoo chimpanzee consortium: An attempt to create an ecologically and socially acceptable habitat. Int. Zoo Yearb..

[36] Anestis S.F. (2005). Behavioral style, dominance rank, and urinary cortisol in young chim-panzees (*Pan troglodytes*). Behaviour.

[37] Sapolsky R.M., Ray J.C. (1989). Styles of dominance and their endocrine correlates among wild olive baboons (*Papio anubis*). Am. J. Primatol..

[38] De Lathouwers M., van Elsacker L. (2004). Comparing maternal styles in bonobos (*Pan paniscus*) and chimpanzees (*Pan troglodytes*). Am. J. Primatol..

[39] Clay L., Paterson M.B.A., Bennett P., Perry G., Phillips C.C.J. (2020). Do Behaviour Assessments in a Shelter Predict the Behaviour of Dogs Post-Adoption?. Animals.

[40] Schuett W., Dall S.R.X. (2009). Sex differences, social context and personality in zebra finches, Taeniopygia guttata. Anim. Behav..

